# Supplementary material for: Whole-Genome Sequencing Reveals the High Nosocomial Transmission and Antimicrobial Resistance of Clostridioides difficile in a Single Center in China, a Four-Year Retrospective Study
Source: Microbiol Spectr. 2022 Jan 12;10(1):e01322-21. doi: 10.1128/spectrum.01322-21 (PMC8754133; doi:10.1128/spectrum.01322-21)
Supplement: SUPPLEMENTAL FILE 1 — Supplemental material. Download SPECTRUM01322-21_Supp_1_seq8.pdf, PDF file, 2.5 MB [file spectrum01322-21_supp_1_seq8.pdf]

Description of Supplementary Files

Fig. S1. ***Clostridium difficile*** **monthly isolation rates between February 2019 to January 2020.**

Fig. S2. ***Clostridium difficile*** **isolation rates from 2019/02 to 2020/01.**

(a) Isolation rates of *C. difficile* isolates correlated with different ages.

(b) Isolation rates of TCD isolates correlated with different ages.

(c) Isolation rates of *C. difficile* isolates from patients with different underlying diseases.

(d) Isolation rates of TCD isolates from patients with different underlying diseases.

Fig. S3. The ward distribution of the samples.

(a) Wards distribution of the 857 fecal samples collecting from inpatient cases.

(b) Wards Distribution of 251 *C. difficile* isolates separated from inpatient cases.

Fig. S4. ***Clostridium difficile*** **isolation rates among different wards.**

The wards collecting less than 20 fecal samples were classified as other wards.

Fig. S5. The nucleotide and amino acid sequences of the truncated toxin-associated genes.

The nucleotide and AA positions causing frameshift mutations are boxed in blue rectangles, and the positions forming stop codons are boxed in green rectangles.

Fig. S6. The transmission network of CDI and CDAC cases based on SNVs.

Only 30 multiple cases clusters were shown in this figure. The red nodes represent CDI cases, and the blue nodes represent CDAC cases. Nodes with pairwise SNVs ≤3 are connected with lines. The shape of arrows represent the sample interval: solid line (in 28d), dashed line (28d to 365d), and vertical slash (>365). The arrowhead point to the later collected isolates.

Fig. S7. The relationship between genetic linkages and epidemiological linkages.

(a) SNVs between each case and the most genetically-closed previous case. (b) The percentages of the epidemiologic-linked case pairs in genetically-linked case pairs according to the different SNV thresholds (from SNV=0 to SNV$\leqslant$10). M68 was used as the reference genome.

Fig. S8. Frequency of the pairwise SNVs from the same MLST.

MLSTs containing less than 10 isolates were not shown in this figure.

Fig. S1

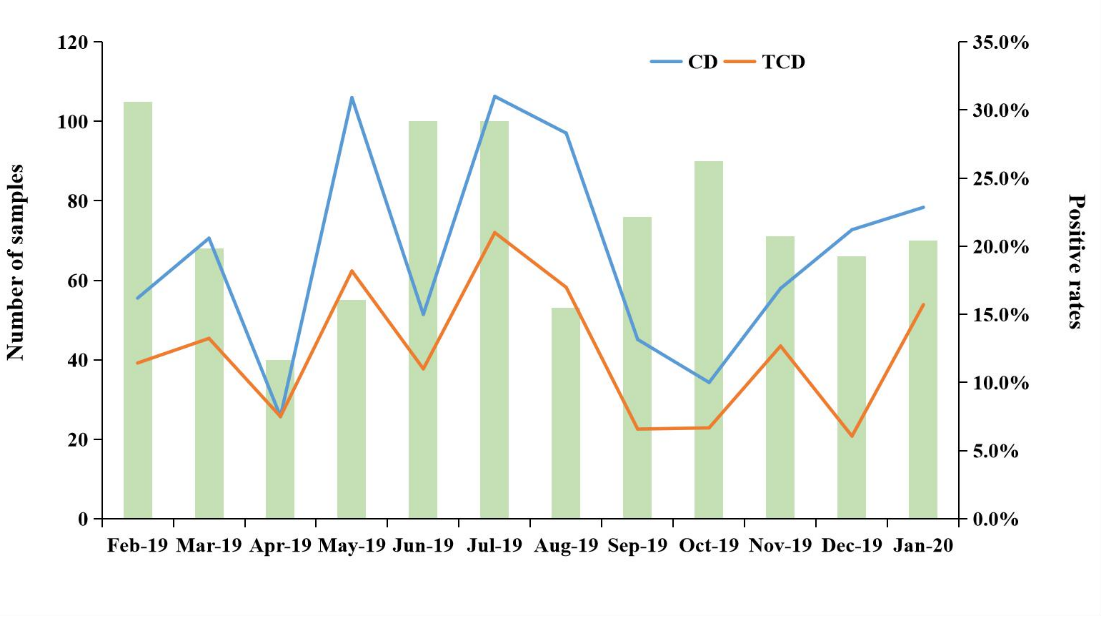
CD
TCD
Number of samples
Positive rates
120
100
80
60
40
20
0
35.0%
30.0%
25.0%
20.0%
15.0%
10.0%
5.0%
0.0%
Feb-19 Mar-19 Apr-19 May-19 Jun-19 Jul-19 Aug-19 Sep-19 Oct-19 Nov-19 Dec-19 Jan-20

Fig. S2

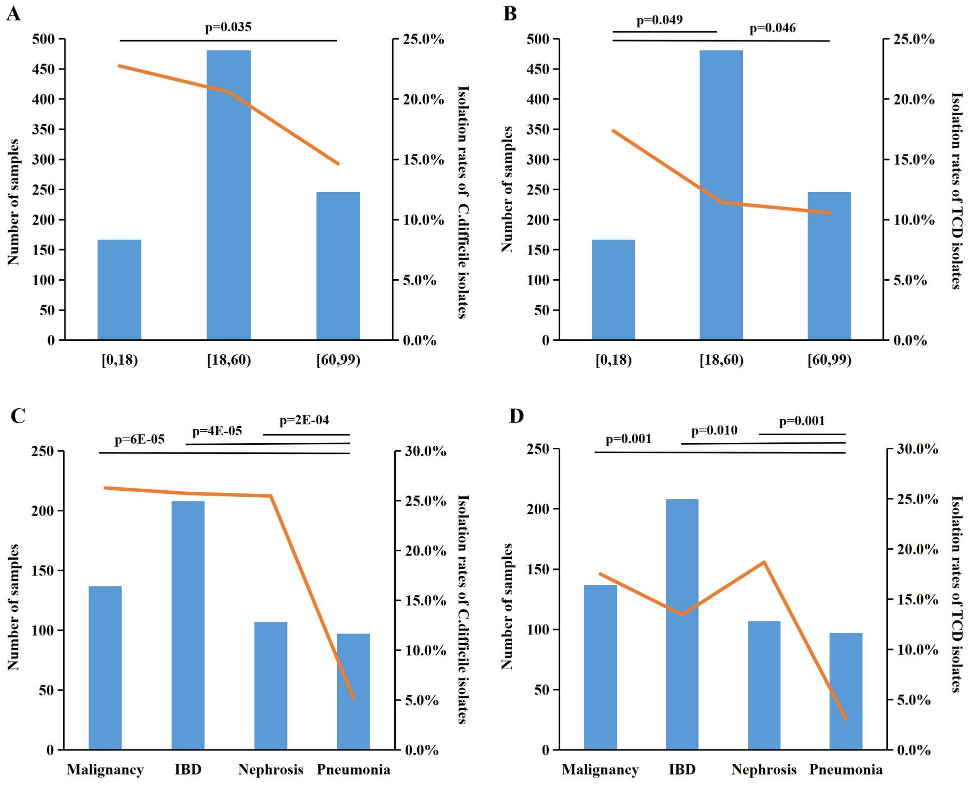
A
p=0.035
Number of samples
Isolation rates of C.difficile isolates
[0,18)
[18,60)
[60,99)
B
p=0.049
p=0.046
Number of samples
Isolation rates of TCD isolates
[0,18)
[18,60)
[60,99)
C
p=6E-05
p=4E-05
p=2E-04
Number of samples
Isolation rates of C.difficile isolates
Malignancy
IBD
Nephrosis
Pneumonia
D
p=0.001
p=0.010
p=0.001
Number of samples
Isolation rates of TCD isolates
Malignancy
IBD
Nephrosis
Pneumonia

Fig. S3

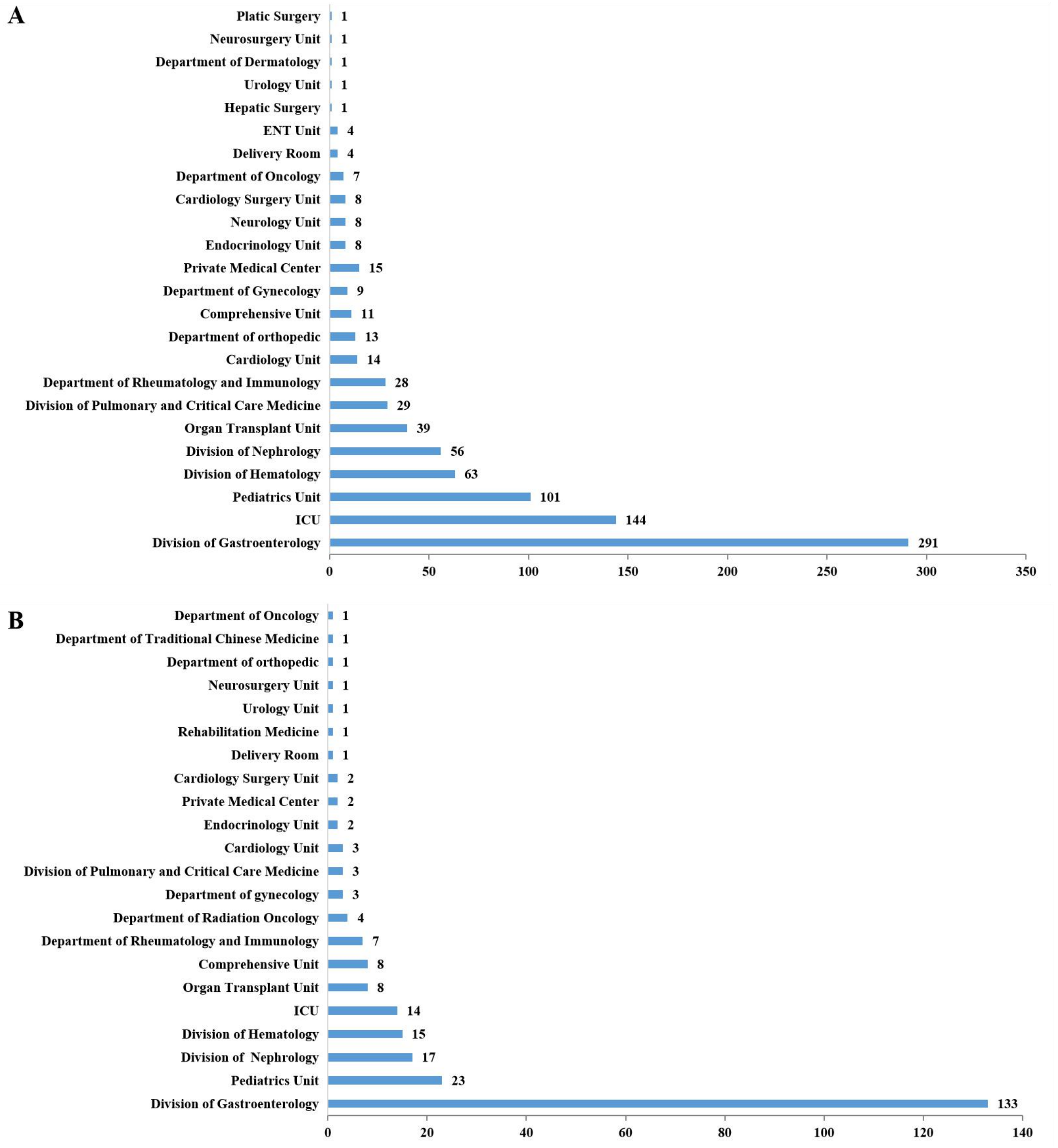
A
Platic Surgery 1
Neurosurgery Unit 1
Department of Dermatology 1
Urology Unit 1
Hepatic Surgery 1
ENT Unit 4
Delivery Room 4
Department of Oncology 7
Cardiology Surgery Unit 8
Neurology Unit 8
Endocrinology Unit 8
Private Medical Center 15
Department of Gynecology 9
Comprehensive Unit 11
Department of orthopedic 13
Cardiology Unit 14
Department of Rheumatology and Immunology 28
Division of Pulmonary and Critical Care Medicine 29
Organ Transplant Unit 39
Division of Nephrology 56
Division of Hematology 63
Pediatrics Unit 101
ICU 144
Division of Gastroenterology 291
0 50 100 150 200 250 300 350
B
Department of Oncology 1
Department of Traditional Chinese Medicine 1
Department of orthopedic 1
Neurosurgery Unit 1
Urology Unit 1
Rehabilitation Medicine 1
Delivery Room 1
Cardiology Surgery Unit 2
Private Medical Center 2
Endocrinology Unit 2
Cardiology Unit 3
Division of Pulmonary and Critical Care Medicine 3
Department of gynecology 3
Department of Radiation Oncology 4
Department of Rheumatology and Immunology 7
Comprehensive Unit 8
Organ Transplant Unit 8
ICU 14
Division of Hematology 15
Division of Nephrology 17
Pediatrics Unit 23
Division of Gastroenterology 133
0 20 40 60 80 100 120 140

Fig. S4

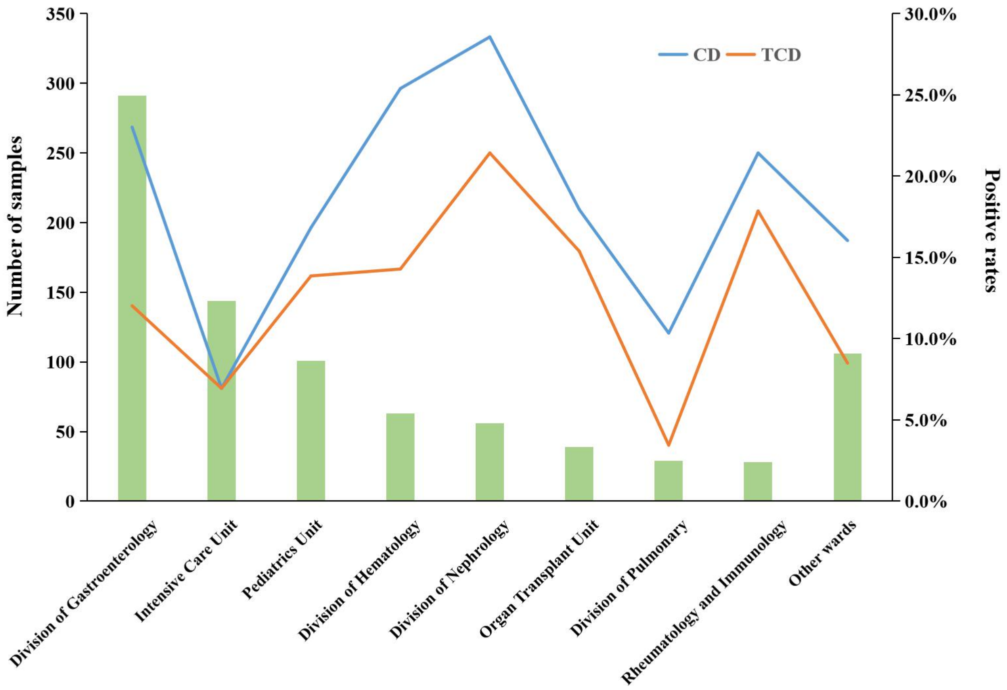
CD
TCD
Number of samples
Positive rates
350
300
250
200
150
100
50
0
30.0%
25.0%
20.0%
15.0%
10.0%
5.0%
0.0%
Division of Gastroenterology
Intensive Care Unit
Pediatrics Unit
Division of Hematology
Division of Nephrology
Organ Transplant Unit
Division of Pulmonary
Rheumatology and Immunology
Other wards

Fig. S5

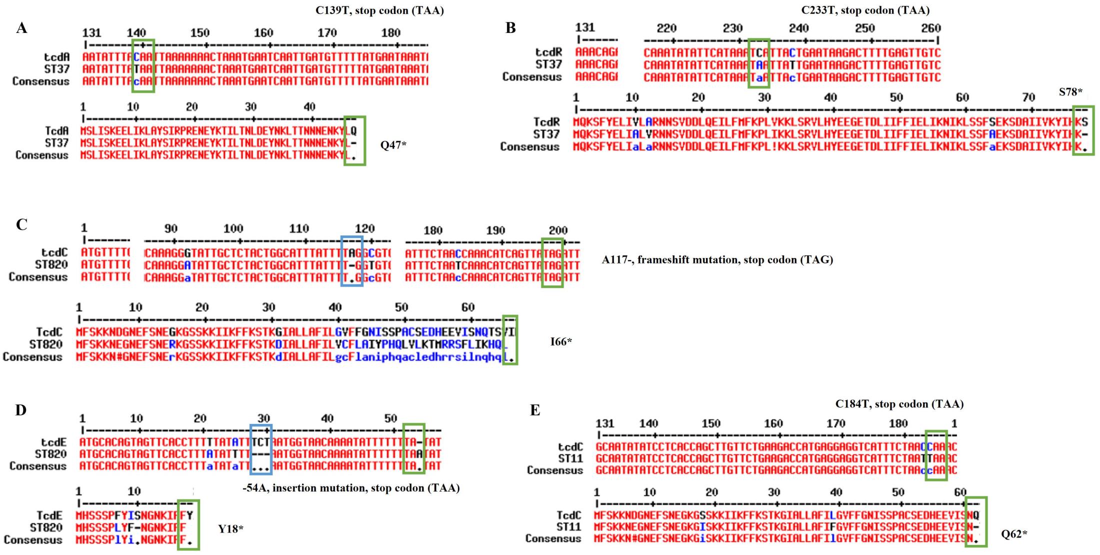
A
C139T, stop codon (TAA)
tcdA
ST37
Consensus
TcdA
ST37
Consensus
Q47*
B
C233T, stop codon (TAA)
tcdR
ST37
Consensus
TcdR
ST37
Consensus
S78*
C
A117-, frameshift mutation, stop codon (TAG)
tcdC
ST820
Consensus
TcdC
ST820
Consensus
I66*
D
-54A, insertion mutation, stop codon (TAA)
tcdE
ST820
Consensus
TcdE
ST820
Consensus
Y18*
E
C184T, stop codon (TAA)
tcdC
ST11
Consensus
TcdC
ST11
Consensus
Q62*

Fig. S6

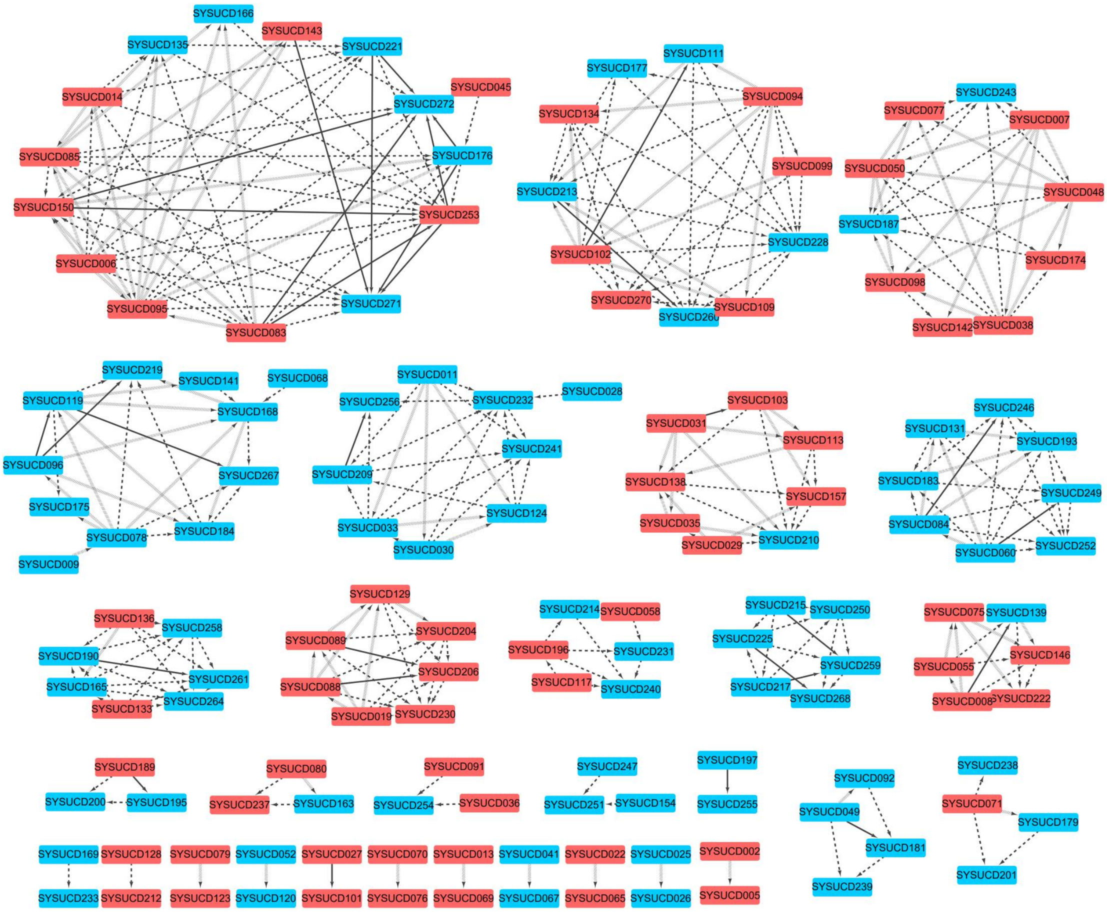
SYSUCD166
SYSUCD143
SYSUCD135
SYSUCD221
SYSUCD045
SYSUCD014
SYSUCD272
SYSUCD176
SYSUCD085
SYSUCD150
SYSUCD253
SYSUCD006
SYSUCD271
SYSUCD095
SYSUCD083
SYSUCD111
SYSUCD177
SYSUCD094
SYSUCD134
SYSUCD099
SYSUCD213
SYSUCD228
SYSUCD102
SYSUCD270
SYSUCD109
SYSUCD260
SYSUCD243
SYSUCD077
SYSUCD007
SYSUCD050
SYSUCD048
SYSUCD187
SYSUCD174
SYSUCD098
SYSUCD142
SYSUCD038
SYSUCD219
SYSUCD141
SYSUCD068
SYSUCD119
SYSUCD168
SYSUCD096
SYSUCD267
SYSUCD175
SYSUCD078
SYSUCD184
SYSUCD009
SYSUCD011
SYSUCD256
SYSUCD232
SYSUCD028
SYSUCD241
SYSUCD209
SYSUCD124
SYSUCD033
SYSUCD030
SYSUCD103
SYSUCD031
SYSUCD113
SYSUCD138
SYSUCD157
SYSUCD035
SYSUCD029
SYSUCD210
SYSUCD246
SYSUCD131
SYSUCD193
SYSUCD183
SYSUCD249
SYSUCD084
SYSUCD060
SYSUCD252
SYSUCD136
SYSUCD258
SYSUCD190
SYSUCD261
SYSUCD165
SYSUCD264
SYSUCD133
SYSUCD129
SYSUCD089
SYSUCD204
SYSUCD206
SYSUCD088
SYSUCD019
SYSUCD230
SYSUCD214
SYSUCD058
SYSUCD196
SYSUCD231
SYSUCD117
SYSUCD240
SYSUCD215
SYSUCD250
SYSUCD225
SYSUCD259
SYSUCD217
SYSUCD268
SYSUCD075
SYSUCD139
SYSUCD055
SYSUCD146
SYSUCD008
SYSUCD222
SYSUCD189
SYSUCD200
SYSUCD195
SYSUCD080
SYSUCD237
SYSUCD163
SYSUCD091
SYSUCD254
SYSUCD036
SYSUCD247
SYSUCD251
SYSUCD154
SYSUCD197
SYSUCD255
SYSUCD092
SYSUCD049
SYSUCD181
SYSUCD239
SYSUCD238
SYSUCD071
SYSUCD179
SYSUCD201
SYSUCD169
SYSUCD233
SYSUCD128
SYSUCD212
SYSUCD079
SYSUCD123
SYSUCD052
SYSUCD120
SYSUCD027
SYSUCD101
SYSUCD070
SYSUCD076
SYSUCD013
SYSUCD069
SYSUCD041
SYSUCD067
SYSUCD022
SYSUCD065
SYSUCD025
SYSUCD026
SYSUCD002
SYSUCD005

Fig.S7

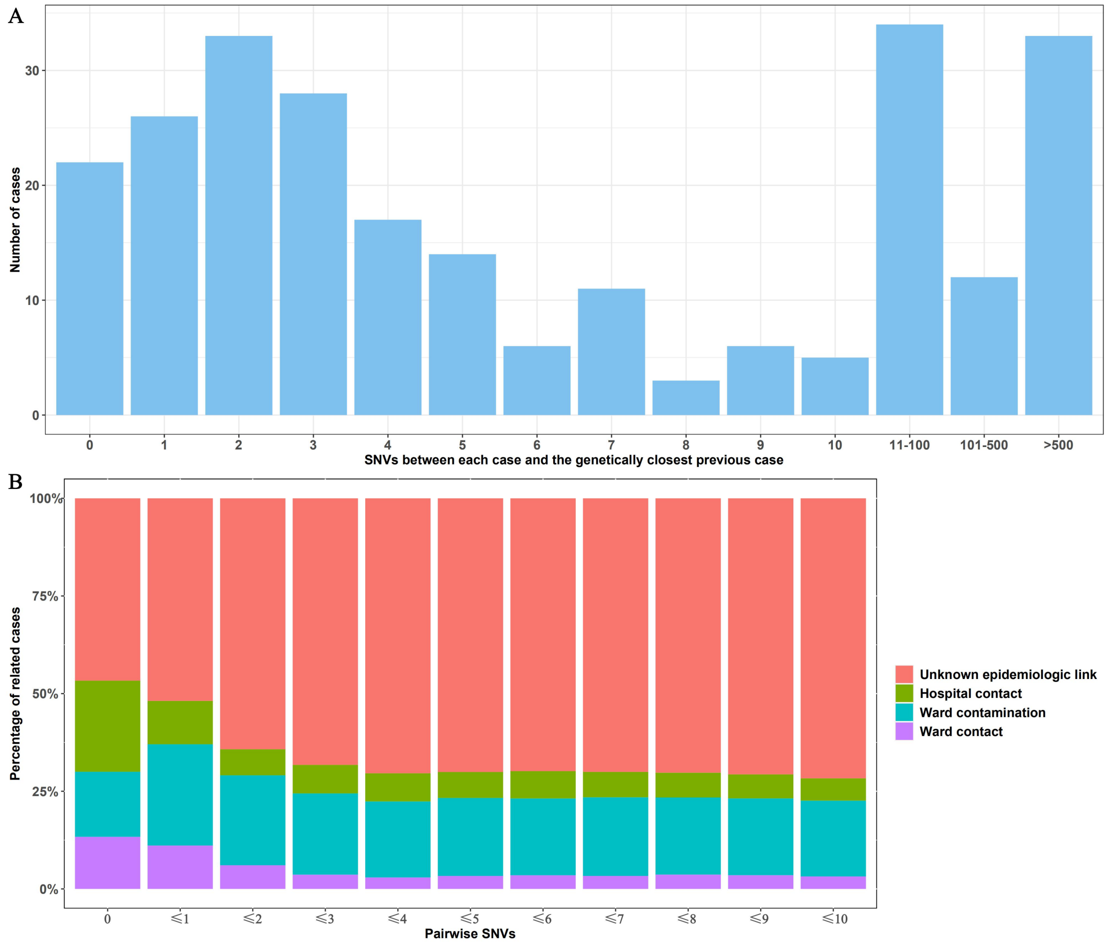
A
Number of cases
0
10
20
30
0
1
2
3
4
5
6
7
8
9
10
11-100
101-500
>500
SNVs between each case and the genetically closest previous case
B
Percentage of related cases
0%
25%
50%
75%
100%
0
⩽1
⩽2
⩽3
⩽4
⩽5
⩽6
⩽7
⩽8
⩽9
⩽10
Pairwise SNVs
Unknown epidemiologic link
Hospital contact
Ward contamination
Ward contact

Fig. S8

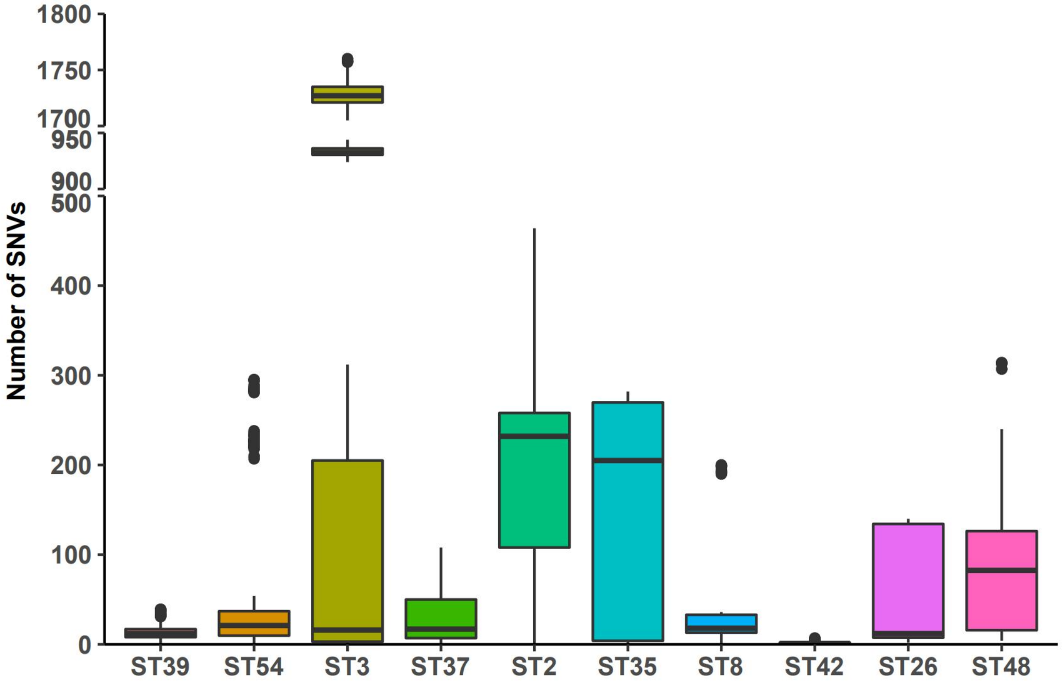
Number of SNVs
0
100
200
300
400
500
900
950
1700
1750
1800
ST39
ST54
ST3
ST37
ST2
ST35
ST8
ST42
ST26
ST48

Table. S1. Clinical characteristics of all the screened patients.

	Total	CD positive	TCD positive	CD negative
**The number of cases, N (%)**	894	173 (19.4%)	110 (12.3%)	721 (80.6%)
**Age, y**				
Mean ± SD	42.24±24.05	**38.31±22.60**[a]	38.31±23.93	**43.18±24.31**[a]
Range	0-98	0-87	1-85	0-98
Median [quartiles]	43 [23, 61]	37 [21.5, 56.5]	37 [16.75, 59]	43 [24, 62]
**Sex, N (%)**				
Female	351	70 (19.9%)	48 (13.7%)	281 (80.1%)
Male	543	103 (19.0%)	62 (11.4%)	440 (81.0%)
**Wards, N (%)**				
Division of Gastroenterology	291	67 (23.0%)	35 (12.0%)	224 (77.0%)
Intensive Care Unit	144	10 (6.9%)	10 (6.9%)	134 (93.1%)
Pediatrics Unit	101	17 (16.8%)	14 (13.9%)	84 (83.2%)
Division of Hematology	63	16 (25.4%)	9 (14.3%)	47 (74.6%)
Division of Nephrology	56	16 (28.6%)	12 (21.4%)	40 (71.4%)
Organ Transplant Unit	39	7 (17.9%)	6 (15.4%)	32 (82.1%)
Division of Pulmonary	29	3 (10.3%)	1 (3.4%)	26 (89.7%)
Rheumatology and Immunology	28	6 (21.4%)	5 (17.9%)	22 (78.6%)
Other wards [b]	106	17 (16.0%)	9 (8.5%)	89 (84.0%)
Outpatients	37	14 (37.8%)	9 (24.3%)	23 (62.2%)
**Underlying diseases, N (%)**				
Malignancy	137	36 (26.3%)	24 (17.5%)	101 (73.7%)
IBD	208	53 (25.5%)	28 (13.5%)	155 (74.5%)
Nephrosis	107	27 (25.2%)	20 (18.7%)	80 (74.8%)
Pneumonia	97	5 (5.2%)	3 (3.1%)	92 (94.8%)
Others	345	52 (15.1%)	35 (10.1%)	293 (84.9%)

Note: IBD (inflammatory bowel diseases). The patients with *C. difficile* were older than without *C. difficile* (p = 0.022)[a]. The wards collecting less than 20 fecal samples were classified as other wards.
